# Light Management in Flexible Glass by Wood Cellulose Coating

**DOI:** 10.1038/srep05842

**Published:** 2014-07-28

**Authors:** Zhi-Qiang Fang, Hong-Li Zhu, Yuan-Yuan Li, Zhen Liu, Jia-Qi Dai, Colin Preston, Sean Garner, Pat Cimo, Xin-Sheng Chai, Gang Chen, Liang-Bing Hu

**Affiliations:** 1 State Key Laboratory of Pulp and Paper Engineering, South China University of Technology, Guangzhou 510640, Guangdong, P.R China; 2 Department of Materials Science and Engineering, University of Maryland College Park, College Park, Maryland, 20742; 3 Corning Incorporated, Corning, NY 14831, United States; 4 These authors contributed equally to this work.

## Abstract

Ultra-thin flexible glass with high transparency is attractive for a broad range of display applications; however, substrates with low optical haze are not ideal for thin film solar cells, since most of the light will go through the semiconductor layer without scattering, and the length of light travelling path in the active layer is small. By simply depositing a layer of TEMPO (2,2,6,6-tetramethylpiperidine-1-oxyl radical)-oxidized wood fibers (TOWFs), we are able to tailor the optical properties of flexible glass dramatically from exhibiting low haze (<1%) to high haze (~56%) without compromising the total forward transmittance (~90%). The influence of the TOWFs morphology on the optical properties of TOWFs-coated flexible glass is investigated. As the average fiber length decreases, the transmission haze of TOWF-coated flexible glass illustrates a decreasing trend. Earth-abundant natural materials for transparent, hazy, and flexible glass have tremendous applicability in the fabrication of flexible optoelectronics with tunable light scattering effects by enabling inexpensive and large-scale processes.

Optically transparent and mechanically flexible electronics have generated significant interest in the past decade due to their ability to meet the increasing technological demands for next-generation electronics^1^,^2^,^3^,^4^,^5^. Emerging flexible glass substrates with a thickness as small as 50 μm exhibit high transparency, superior surface quality, thermal stability, chemical compatibility, and mechanical flexibility that enable displays, photovoltaics, lighting, and touchscreens that are fabricated by roll-to-roll methods^6^,^7^,^8^,^9^,^10^,^11^,^12^. Transparent films with high diffuse light scattering have potential applications in solar cells, OLED light systems, backlight units of liquid crystal displays (LCD), signage, etc. In general, there are two types of methods (surface relief and particle coating) widely used to make transparent films with high light scattering properties^13^,^14^,^15^,^16^,^17^. Light diffuse scattering films based on surface relief have a microstructural surface with a tunable shape and a high optical transmittance and are normally fabricated from plastic films or plates, but this involves complicated and time-consuming fabrication processes and expensive equipment^18^,^19^. Particle diffuse scattering is the primary method to produce diffusive films with both high transparency and high haze due to its relatively simple processing procedure; however, non-uniform distribution of particles in the coating layer is still an obstacle that will affect luminance uniformity of the optical diffuser^19^. In general, roughening the glass surface is realized by wet or dry etching or mechanical treatments like sandblasting. These methods for structuring the glass surface tend to cause damage, cracks or defects, and line textures at the surface^20^.

Using natural materials to fabricate transparent, hazy, and flexible glass without damaging the glass surface by a scalable and simple method is attractive. Wood pulp, primarily constituting cellulose, is an earth-abundant, renewable, light-weight, and biodegradable natural material. Paper made of wood pulp displays a wide range of properties due to the hierarchical structure of wood fiber; it may be transparent or opaque, porous or hermetic, rough or ultra-smooth, flexible or rigid, fragile or tough^21^,^22^,^23^. To achieve desirable properties of paper, mechanical refining and chemical modifications are applied to alter the morphology and surface properties of wood fibers. TEMPO(2,2,6,6-tetramethylpiperidine-1-oxyl radical)-mediated oxidation is generally considered to be one of the most efficient methods to modify the wood fibers^24^,^25^. By introducing carboxyl groups in the C6 of cellulose during TEMPO oxidation, wood fibers tend to easily collapse and form a densely packed fiber network due to weak interfibrillar hydrogen bonds in the fiber cell wall induced by strong electrostatic repulsive forces.

It has been verified that the diameter of the fiber has a significant effect on the optical properties of paper by our group^26^. Through a combination of TEMPO-treatment and homogenization of wood fibers, the fiber diameter is modulated from 25 μm to 10 nm resulting in a dramatic decrease of the transmission haze of paper from 77% to 20% at a wavelength of 550 nm^26^. In addition, a novel nanostructured transparent paper with both high transparency (~96% at 550 nm) and high transmission haze (60% at 550 nm) was developed by our group using TEMPO-oxidized wood pulp recently^27^. TEMPO-treated wood pulp is inclined to form dense fiber network during the fabrication procedure, permitting more light to pass through; moreover, larger fiber diameter renders stronger light scattering behavior of fabricated transparent paper. These results attained in our lab allow us to fabricate flexible glass with light management properties using natural materials and pave a new way to apply TEMPO-oxidized wood fibers (TOWF) in the field of transparent and flexible optoelectronics with a simple and low cost method.

In this study, we firstly use TEMPO-oxidized wood pulp to produce a flexible, transparent, and controllable hazy glass through a simple and scalable deposition process. This TOWFs-coated flexible glass presents the ability to manage light propagation while maintaining original optical transmittance. Also, an optically transparent flexible glass with tailored transmission haze is fabricated by mechanically altering the morphology of TEMPO-treated wood pulp. This not only exhibits both high transparency and controllable haze, but also shows strong bonding between the fiber layer and flexible glass, which has great potential for applications in flexible optoelectronics.

## Results

**Figure 1:**
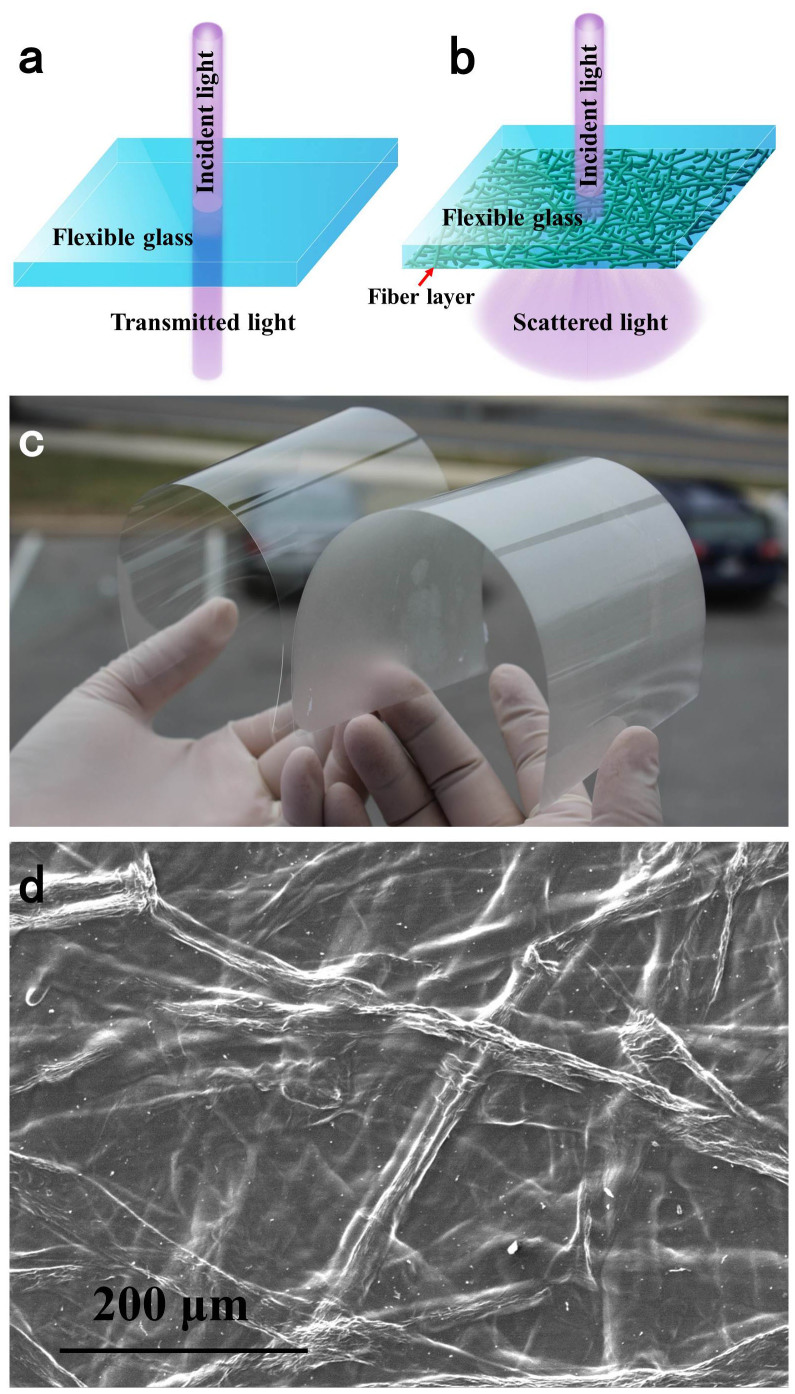
Schematic showing (a) the incident light passes through flexible glass and (b) TOWF-coated flexible glass; (c) digital images of flexible glass (left, thickness = 100 μm) and long TOWFs-coated flexible glass (right, thickness = 127 μm); (d) top-view SEM image of TOWFs layer.

Glass (Corning® Willow® Glass) with a thickness of ≤200 μm illustrates a high transparency and mechanical flexibility^11^,^28^,^29^. When a beam of light strikes the surface of flexible glass, the incident light directly propagates through without changing the angular direction as shown in Figure 1a; however, some applications require the flexible glass to scatter the transmitted light (high haze) while perpetuating a high optical transmittance. To achieve this goal, we strategically fabricated a flexible glass possessing both excellent transmittance and high transmission haze by depositing a layer of TOWFs with a thickness of about 27 μm. The transmitted light is significantly scattered by the transparent thin layer of TOWFs due to the large fiber diameter, and forms a larger illuminated circle than that of flexible glass (Figure 1b). Figure 1c displays the appearance and flexibility of glass and TOWFs-coated glass. To understand the structure of the TOWFs layer, a SEM was used to observe the network of TOWFs on flexible glass. Collapsed TOWFs randomly stack together and form a dense TOWFs layer on flexible glass, in which no obvious micro-cavities within the TOWF layer are observable (Figure 1d). The excellent packing density of the TOWF layer allows more incident light to pass through the TOWF-coated flexible glass without sacrificing the inherent optical transmittance of flexible glass. The principle for high transparency of TOWFs layer was described in detail in our previous work^27^.

### Morphologies of TEMPO-oxidized wood pulp

To produce highly hazy TOWFs-coated flexible glass without compromising high transparency of flexible glass, a TEMPO/NaClO/NaBr solution was utilized to introduce carboxyl group in the C6 of cellulose to increase the electrostatic repulsive force between neighboring cellulose nanofibers in fiber cell wall. With the assistance of mechanical stirring, the wood fibers tend to be cleaved in the axial direction that reduces fiber length and part of wood fibers break into small fiber debris. The reduced fiber length, dramatic increased fine content (fiber length < 0.2 mm), and the easily crushed hollow structure of fiber due to weak bonds between neighboring cellulose nanofibers lead to form high density of fiber network that contributes to the high transparency of TOWFs-coated flexible glass (as shown in Table 1).

**Figure 2:**
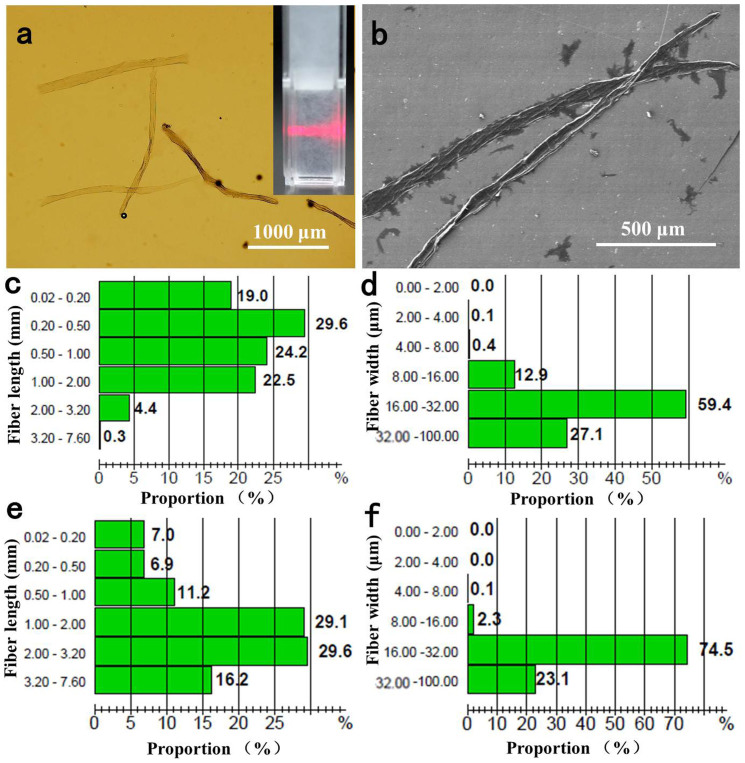
(a) Microscopic image and (b) SEM image of long TOWFs; inset indicates a red laser passes through TOWF suspension (0.5 wt %) from the left side; (c) length fraction and (d) width fraction of long TOWFs; (e) length fraction and (f) width fraction of original wood fibers.

The morphology of TOWFs was obtained using an optical microscope (OLYMPUS BX51). Figure 2a shows the morphology of TOWFs illustrating the wood fibers are cleaved in the axial direction and no obvious fibrillated wood fibers are observed. The inset in Figure 2a displays the visual appearance of long TOWFs suspension with a concentration of 0.5 wt %. The suspension was irradiated by a laser beam with a wavelength of 650 nm to illustrate the light scattering effect of TOWFs. As we see from the SEM image of long TOWFs in Figure 2b, the wood fibers are unzipped during the TEMPO oxidation and tend to easily collapse during the drying process. This results in much higher packing density in the TOWFs layer on flexible glass that is contributed to the high transparency. To quantify the morphology of TOWFs, a Kajaani FS300 fiber analyzer was used to measure the length and width distribution of long TOWFs. The average length and width of the long TOWFs is around 0.72 mm and 26 μm, respectively, and the fine content is approximately 19%. The length distribution of long TOWFs and original wood fibers are displayed in Figure 2c and Figure 2e, respectively. The wood fibers become short fibers with a length of less than 1.0 mm due to TEMPO oxidation and mechanical stirring, and the fine content of wood pulp increases dramatically, approximates accounting for 19% based on total fibers. Figure 2d indicates the width distribution of the TOWFs in comparison to original fibers (Figure 2f). The numbers of wood fibers with a fiber width ranging from 16 to 32 μm demonstrates a declining trend, but for the wood fibers with a fiber width ranging from 8–16 μm and 32–100 μm, slight increments on the portion of wood fiber are obtained. The packing density of TOWF film on flexible glass is inclined to increase due to increased short fibers, high fine content, and easily collapsed TOWF.

### Optical properties of TOWF-coated flexible glass

**Figure 3:**
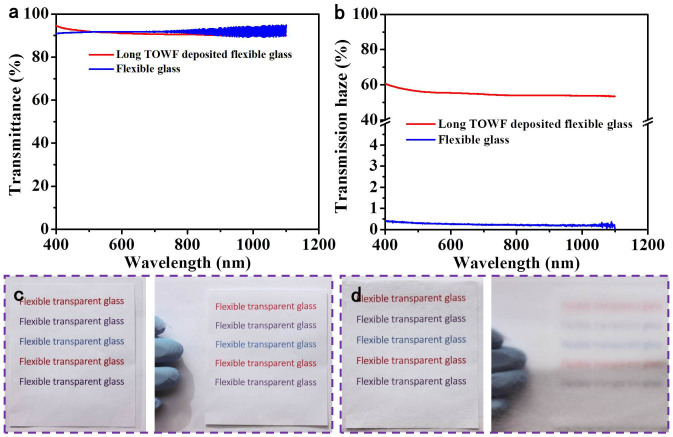
(a) Optical transmittance and (b) transmission haze of long TOWFs-coated flexible glass; digital image showing the transmission haze of (c) flexible glass and (d) long TOWFs-coated flexible glass (left: both closely contact underneath color letters, right; both at a distance of 1.5 cm from underneath color letters.

To fabricate the coated substrate samples, TOWF suspension was manually deposited on one side of 100 μm thick Corning® Willow® Glass flexible substrate using a pipette to fabricate transparent, hazy, and flexible glass. A variety of solution coating methods are suitable to apply the TOWF layer onto the flexible glass, and the specific process can be chosen based on the overall fabrication and through-put requirements. UV-Vis spectrometer measurements with an integrating sphere (PerkinElmer® USA) were used to measure the optical transmittance and transmission haze of TOWFs-coated flexible glass. Figure 3a is the optical transmittance of flexible glass and TOWFs-coated flexible glass. The transmittance curve of flexible glass and the TOWF-coated flexible glass are almost overlapping in the range from 1100 nm to 400 nm, which illustrates the TOWF layer coated on flexible glass has a high optical transparency and thus does not inhibit the optical transmittance characteristic of flexible glass.

Another significant optical property introduced is the high transmission haze after TOWF deposition. As shown in Figure 3b, long TOWF-coated flexible glass has a transmission haze of ~56% at a wavelength of 550 nm. Figure 3c shows the flexible glass with low transmission haze. The underneath letters are distinctly observable as one piece of flexible glass covers over it (left image in Figure 3c). With a separation of about 1.5 cm between the flexible glass and the underneath pattern, the letters still look very clear due to the low haze of flexible glass. However, for the long TOWF-coated flexible glass, it shows a different phenomenon. The letters are clearly observable as the underneath paper with printed letters contacts it closely (left image in Figure 3d). With a separation of about 1.5 cm between the long TOWF-coated flexible glass and the pattern, the underneath pattern becomes quite obscure due to the intensive light scattering effect (right image in Figure 3d).

**Figure 4:**
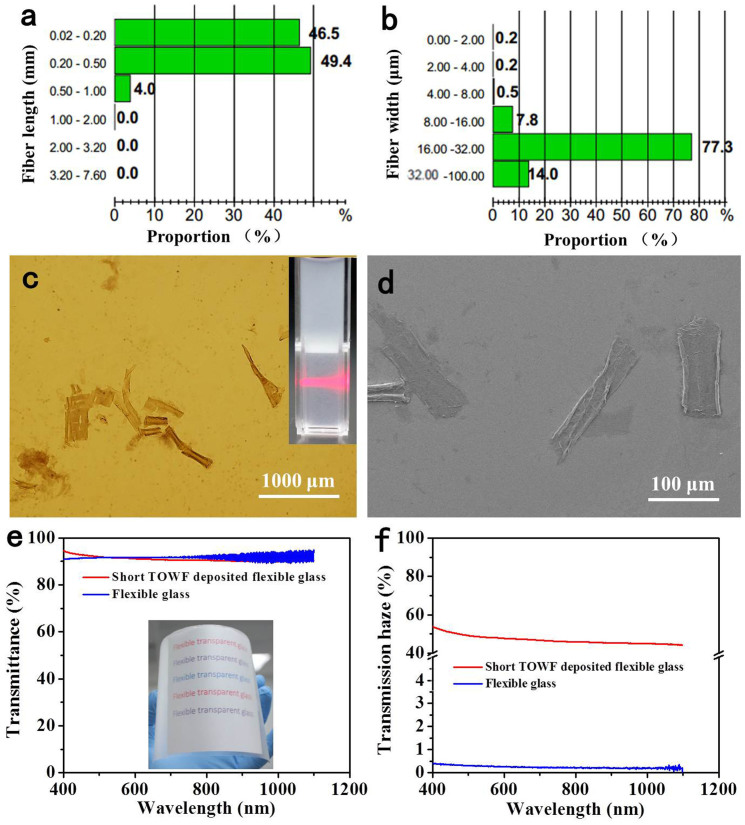
(a) Length fraction and (b) width fraction of short TOWFs. (b) Microscopic image and (c) SEM image of TEMPO-oxidized short wood fibers (TOWFs); inset shows a red laser passes through short TOWFs suspension (0.5 wt %) from the left side with regard to the viewing perspective. (d) Optical transmittance and (e) transmission haze of short wood fibers-coated flexible glass. (Inset in figure 4c: short wood fibers suspension, inset in figure 4e shows wood fiber-coated flexible glass.

In addition, the influence of the TOWFs morphology on the optical properties of TOWFs-coated flexible glass was also studied. A TOWFs suspension was continually stirred in a magnetic stirrer at a speed of 700 rpm for another 10 h to mechanically cut off wood fibers. The histogram for the length and width distribution of short TOWFs was displayed in Figure 4a and 4b. About 96% of total TOWFs has a fiber length of less than 0.5 mm and the width of most TOWFs is in the range of 16 ~ 32 μm. As shown in the Table 1, both long and short TOWFs have similar fiber width, however, the short TOWFs presents a shorter fiber length and higher fine content than long TOWFs. Figure 4c shows the morphology of short TOWFs. Wood fibers were cleaved into smaller sized fibers in the axial direction and became cracked under mechanical stirring due to the breakage of the weak interfibrillar bonds. The short TOWF suspension seems to be more homogenerous compared to a TOWF suspension without additional stirring (see insert in Figure 4c) in term of visual appearance, and a red laser propagating through the suspension (0.5 wt %) is gradually scattered from the left side with regard to the viewing perspective. A SEM image of short-TOWFs displayed in Figure 4d shows ribbon-like fibers were formed after drying, which is beneficial to form a dense TOWFs layer. The optical transmittance of short TOWFs-coated flexible glass is similar to that of bare flexible glass (as shown in Figure 4e). The inset in Figure 4e illustrates short TOWF-coated flexible glass over a printed pattern. The transmission haze is approximately 48% at a wavelength of 550 nm which is slightly lower than that of long TOWF-coated flexible glass (Figure 4f). A previous simulation has shown that the fiber diameter plays a significant role in the light scattering of nanopaper^26^. In this case, the average width of short TOWF is almost the same as that of long TOWF (Table 1), therefore, the change in optical haze across different TOWF is likely due to the morphology difference. TOWFs layer is a mixture of fibers and fines. The light scattering behavior of TOWFs layer is thus largely related to the fine content. In comparison with TOWFs, the fiber debris has a relatively smaller dimension that causes lower scattering. As shown in Table 2, the fine content of short TOWFs suspension is about twice higher than that of long TOWFs that renders the slightly lower haze of short TOWFs layer.

### Mechanical properties of TOWF-coated flexible glass

**Figure 5:**
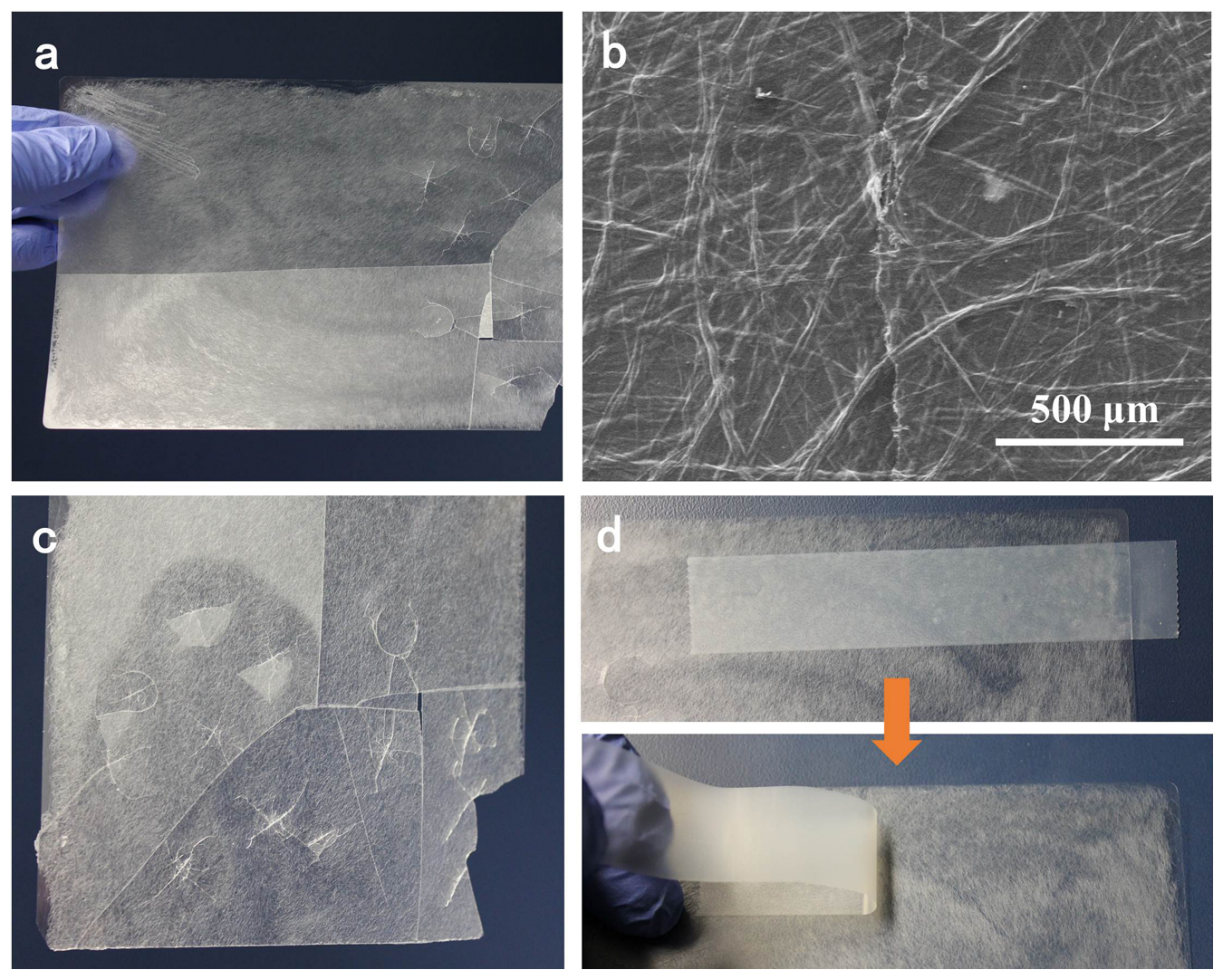
(a) Digital photo of fractured long TOWFs-coated flexible glass, (b) an SEM image of fractures; (c) long TOWFs-coated flexible glass showing a decrease of crack propagation, and (d) adhesion testing between long TOWFs layer and flexible glass.

Particle coatings are widely used to improve the light scattering effect for solar cell applications^30^,^31^,^32^. TOWFs have a network structure that is different from particles, which leads to a large mechanical strength of 105 MPa^27^. TOWFs simultaneously show a strong binding energy with the glass substrate, and can also hold glass pieces together, which can be extremely useful in certain applications. The long TOWFs-coated flexible glass presents the ability to keep the coated substrate structure intact. As shown in Figure 5a and 5c, the TOWFs-coated flexible glass fractures upon impact, but the cracked flexible glass fragments still remain in place. To deeply understand the mechanism for the coherent coating performance of TOWFs-coated flexible glass, a SEM was further used to observe the fractures. The SEM image in Figure 5b shows the fracture of TOWFs-coated flexible glass is connected by TOWFs, which keep the fractured glass fragments integrated. The adhesion strength between the TOWFs layer and flexible glass was evaluated using a tape test. Figure 5d shows the testing procedure. A piece of tape was attached to the TOWFs layer and pressed several times to make sure the tape strongly adhered, then the tape was detached with a peeling speed of roughly 3 cm/s. No fibers are observed in the peeled tape, demonstrating the strong bonding strength between the TOWFs layer and flexible glass. We also bent the coated glass to a small radius, ~120 mm, and no delamination of TOWFs from the glass was observed.

## Discussion

High light scattering enhances the light trapping in solar cells due to the increase of the path length of light in the active layer, resulting in increased power conversion efficiency (PCE) of thin film solar cells^27^,^33^,^34^,^35^,^36^. In our previous works, we successfully applied our transparent paper made of long TOWFs on organic solar cells via a simple attachment, showing an improved PCE of ~10%^27^. This enhancement is attributed to better light harvesting from the diffused light. Furthermore, a paper-based GaAs solar cell was demonstrated by simply laminating transparent paper fabricated from long TOWFs to the top of solar cell using polyvinyl alcohol (PVA) as glue. This paper-based anti-reflection coating for photovoltaics presents an angle insensitive property and an increased PCE of 23.91%^36^. Transparent paper exhibits intense light scattering behavior, but wood fibers made of cellulose are quite hygroscopic, which affects the stability of the optical properties of TOWFs layer. Depositing long TOWFs on glass substrates instead of directly producing transparent paper indicates a path towards industrial applications of dense TOWF networks for photovoltaics.

**Figure 6:**
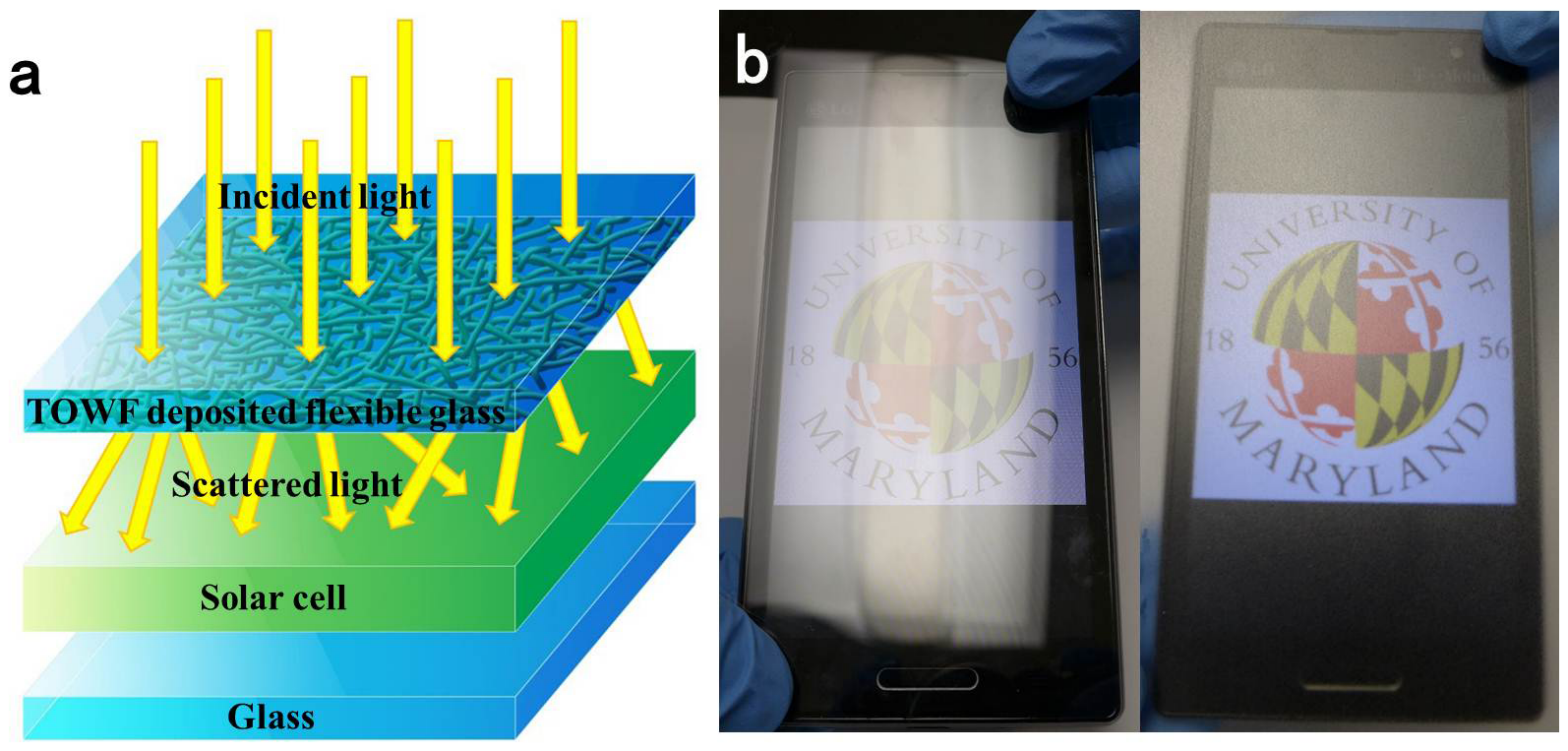
(a) Schematic of solar cells with a top layer of TOWF-coated flexible glass to enhance light trapping performance; (b) flexible glass (left) and short TOWF-coated flexible glass (right) covered on mobile phone screen shows anti-glare performance.

For most solar cell installations, glass substrates are used to encapsulate the cell to protect the device from environmental hazards (moisture, debris, etc.). A TOWF coating can supply a light scattering effect to the existing encapsulation glass. Moreover, due to the UV filtering ability of glass, TOWFs under glass will have much improved UV-vis lifetime than bared paper film. A schematic showing the potential application of TOWFs-coated flexible glass on the encapsulation of solar cell is shown in Figure 6a. The TOWFs layer facing the solar cell is adequately protected by flexible glass. Incident light is scattered by the TOWFs layer towards the active layer of the solar cell, which efficiently enhances the power conversion efficiency by increasing path length of light.

Highly transparent substrates with different light scattering properties can be used for various purposes. Highly hazy substrates are suited for the fabrication of photovoltaic devices with enhanced light trapping ability^37^,^38^,^39^; while substrates with an optical haze of <1% is desirable for displays^40^,^41^,^42^,^43^; and appropriately hazy substrates can endow outdoor electronics with anti-glaring property^26^. The short TOWFs-deposited flexible glass demonstrates relatively low light scattering phenomena compared to long TOWFs coated flexible glass, which is suited for displays used in outdoor electronics. Flexible glass (left) and short TOWFs-coated flexible glass (right) were covered on the screen of a mobile phone (Figure 6b) to evaluate the anti-glaring performance under bright ambient atmosphere. The logo of University of Maryland was displayed in the mobile phone. As the mobile phone covered with a piece of flexible glass is exposed to light, it presents glare and the logo underneath looks obscure. The mobile phone covered with short –TOWFs-coated flexible glass, however, exhibits excellent anti-glaring characteristics (right image in Figure 6b) and the logo is quite clear.

In conclusion, we demonstrate a uniform coating of one-dimensional TOWF on a flexible glass substrate that dramatically increases the optical haze from <1% to ~56% without decreasing the total optical transmittance. Such modified glass shows a large anti-glare effect useful for displays. When applied to glass encapsulation layers widely used in current solar cell installations, a TOWF coating increases the light scattering, which offers a higher absorption and solar cell efficiency in a broadband. The 1D cellulose based coating shows superior coherent mechanical strength and excellent mechanical binding with glass substrate, which can improve the durability of flexible glass. Highly transparent flexible glass with tailored light scattering behavior also possesses a potential application in solar cells, displays, and OLED light systems. This transparent, hazy, and flexible glass sheds a light to the fabrication of next-generation flexible electronics in combination with earth-abundant and renewable natural materials.

## Methods

### Preparation of TEMPO-oxidized wood fibers and TOWF coated flexible glass

A bleached sulfate softwood pulp (Southern Yellow Pine) was used to prepare TEMPO-oxidized wood fibers according to the method proposed in our previous literatures^26^,^44^. Residual chemicals were removed by a filtration at the end of TEMPO oxidation. The wood pulp was diluted into a 1% suspension and then stirred at a speed of 1000 rpm for 1 h, which was repeated twice to assure the enough swell of TEMPO-oxidized wood fibers. The short TOWF were prepared by stirring the obtained long TOWF suspension (1%) with a magnetic stirrer at a speed of 700 rpm for 10 h. To get the excellent light scattering behavior, TOWF suspension (1.0%) was dropwise added onto flexible glass with a maximum volume of 80 mL to deposit a thick TOWF layer on flexible glass (10 cm* 20 cm), and it then dried at room temperature for 24 h.

## Figures and Tables

**Table 1:** Dimension of original wood fibers and long TOWFs

	Average width/μm	Average length/mm	Fine content/%
Original wood fibers	27	1.98	7
Long TOWFs	26	0.72	19.0

**Table 2:** Dimension of long TOWFs and short TOWFs

	Average width/μm	Average length/mm	Fine content/%
Long TOWFs	26	0.72	19.0
Short TOWFs	25	0.23	45.5
